# Differentially Expressed in Chondrocytes 2 (DEC2) Increases the Expression of IL-1β and Is Abundantly Present in Synovial Membrane in Rheumatoid Arthritis

**DOI:** 10.1371/journal.pone.0145279

**Published:** 2015-12-28

**Authors:** Juri Olkkonen, Vesa-Petteri Kouri, Joel Hynninen, Yrjö T. Konttinen, Jami Mandelin

**Affiliations:** 1 Department of Medicine, University of Helsinki and Helsinki University Hospital, Helsinki, Finland; 2 ORTON Orthopaedic Hospital of the Invalid Foundation, Helsinki, Finland; CNRS, University of Strasbourg, FRANCE

## Abstract

**Objective:**

Patients with rheumatoid arthritis (RA) have altered circadian rhythm of circulating serum cortisol, melatonin and IL-6, as well as disturbance in the expression of clock genes ARNTL2 and NPAS2. In humans, TNFα increases the expression ARNTL2 and NPAS2 but paradoxically suppresses clock output genes DPB and PER3. Our objective was to investigate the expression of direct clock suppressors DEC1 and DEC2 (BHLHE 40 and 41 proteins) in response to TNFα and investigate their role during inflammation.

**Methods:**

Cultured primary fibroblasts were stimulated with TNFα. Effects on DEC2 were studied using RT-qPCR and immunofluorescence staining. The role of NF-κB in DEC2 increase was analyzed using IKK-2 specific inhibitor IMD-0354. Cloned DEC2 was transfected into HEK293 cells to study its effects on gene expression. Transfections into primary human fibroblasts were used to confirm the results. The presence of DEC2 was analyzed in (RA) and osteoarthritis (OA) synovial membranes by immunohistochemistry.

**Results:**

TNFα increased DEC2 mRNA and DEC2 was mainly detected at nuclei after the stimulus. The effects of TNFα on DEC2 expression were mediated via NF-κB. Overexpression, siRNA and promoter activity studies disclosed that DEC2 directly regulates IL-1β, in both HEK293 cells and primary human fibroblasts. DEC2 was increased in synovial membrane in RA compared to OA.

**Conclusion:**

Not only ARNTL2 and NPAS2 but also DEC2 is regulated by TNFα in human fibroblasts. NF-κB mediates the effect on DEC2, which upregulates IL-1β. Circadian clock has a direct effect on inflammation in human fibroblasts.

## Introduction

Rheumatoid arthritis (RA) is a common chronic inflammatory joint disease. RA patients suffer from chronic fatigue [1]. Pain, joint stiffness and functional disability are most prominent in the morning [2]. These symptoms reflect abnormal circadian rhythms of circulating inflammatory cytokines TNFα [3] and IL-6 as well as serum cortisol in RA [4, 5]. Many physiological and pathological processes are under circadian regulation. A central circadian pacemaker is located in the suprachiasmatic nucleus (SCN) of the hypothalamus [6, 7]. Because circadian rhythm of the SCN is not exactly 24h in humans [8], light adjusts the rhythm of the central pacemaker. The central circadian pacemaker synchronizes the peripheral molecular pacemakers located in all other tissues [6].

The function of the molecular clock is based on rhythmic oscillation of transcription and translation of reciprocal clock genes. Brain and muscle Arnt-like protein-1 (BMAL1 or ARNTL) and Circadian Locomotor Output Cycles Kaput (CLOCK) form a heterodimer which upregulates clock controlled genes by binding to an E-box element in the promoter of the clock controlled genes. Some of the upregulated genes, Periods and Cryptochromes, form the components of the best known negative feedback loop [9]. The clock molecules, DEC1 (BHLHE40) and DEC2 (BHLHE41), form another less known negative feedback loop because they compete with BMAL1/CLOCK for E-box binding [10].

Clock genes are needed for proper immune cell function. Notably, autoimmune diseases develop in aging DEC1 deficient mice which display increased production of IL-4 and IL-10 without affecting IFN-γ [11]. In contrast, its paralogue DEC2 is selectively expressed in Th2 cells and enhances their development in mice leading to improper Th2 responses in asthma and parasite response models [12]. In addition, a connection between circadian clock and arthritis has been described. Arthritis is exacerbated in Cry1 and Cry2 deficient mice [13] and dysfunctional clock is present in RA patients [14, 15]. TNFα affects the clock and in human cells, in contrast to that of mice [16], the upregulated genes are ARNTL2 and NPAS2, functional paralogues of BMAL1 and CLOCK, respectively. Paradoxically, however, TNFα suppresses clock controlled genes DBP and PER3. Thus, we hypothesized that the negative regulators in the molecular clock DEC1, DEC2 or both are affected by TNFα. Because of our hypothesis and their central role in immune cell function, their regulation *in vitro* by TNFα and expression *in vivo* in RA were studied.

## Materials and Methods

### Subjects

The research plan and this study were approved by the ethical committee of the Helsinki University Central Hospital (Dnro 165/E6/03). Written informed consent from each patient was obtained to collect sample for research purposes. Guidelines of the Declaration of Helsinki were followed. RA patients fulfilled the 2010 ACR-EULAR classification criteria of RA [17]. None of the patients were treated with anti-TNF agents or other biologicals. Tissue samples of both RA (n = 6) and OA (n = 5) patients were taken at 10 a.m. ± 2 h during synovectomy or operation for total joint replacement. Samples were formalin fixed and embedded in paraffin.

### Cell culture

Primary human fibroblast cultures (n = 6) were established and characterized as previously described [18]. Briefly, tissue samples were minced into small pieces with a sterile scalpel in a laminar flow hood. The explants were left overnight in RPMI-1640 medium containing 10% fetal bovine serum with 1000 U/ml penicillin and 1 mg/ml streptomycin (10×) solution. The next day, the media were changed to basal RPMI with 10% FBS media and 100 U penicillin and 0.1 mg streptomycin (1× solution). The medium was changed twice a week. The explants were removed until roughly 80% monolayer confluence was reached, and the cells were subcultured 1:3 until confluent. The cells were frozen at passage 2 for subsequent experiments. After thawing, the cells were cultured in RPMI-1640 medium (Lonza Group, Basel, Switzerland) containing 10% fetal bovine serum (FBS; Lonza) 100 IU/ml penicillin and 0.1 mg/ml streptomycin and used in passages 4–5. Stimulation and inhibitor experiments were performed with three different donor fibroblasts. Transfection experiments were performed with single donor fibroblasts. In Amaxa Nucleofector II transfection experiments, fibroblasts were cultured in DMEM medium (Thermo Fisher Scientific, Waltham, USA; cat# 41965) containing 10% FBS (Lonza) with 100 IU/ml penicillin and 0.1 mg/ml streptomycin.

The synchronization of the molecular clock in cells was performed as described elsewhere with [15] with minor modification. Briefly, cultured human primary fibroblasts were seeded on 24-well plates at 4x10^4^ cells per well in RPMI-1640 containing antibiotics and 1% FBS, cultured for 24 h after which the medium in wells was replaced with RPMI-1640 media containing antibiotics, 1% FBS and TNFα, IMD-0354 or DMSO when indicated.

HEK293 cells were cultured in DMEM medium (Thermo Fisher Scientific, cat# 41965) containing 10% FBS (Lonza) with 100 IU/ml penicillin, 0.1 mg/ml streptomycin and 1 mM pyruvate (Lonza, cat# BE13-115E).

### Cell stimulation

Human primary fibroblasts were synchronized as described in the previous section. At t = 0, the media was replaced with RPMI-1640 media containing antibiotics, 1% FBS and TNFα (10 ng/ml; R&D Systems, Minneapolis, USA) or with media containing no added stimulants (negative control). At indicated times, the wells were washed with PBS and cells were lysed with 350 μl RLT lysis buffer (RNeasy kit, Qiagen, Hilden, Germany).

To study the effect of NF-κB inhibition on DEC2 regulation, IKK-2 inhibitor IMD-0354 (cat# I3159; Sigma-Aldrich Corporation, St. Louis, USA) was used. 24 h after plating the cells, the media was replaced with RPMI-1640 containing antibiotics, 1% FBS, and IMD-0354 in a final concentration of 1 μM or DMSO in the same final concentration as was achieved when IMD-0354 (dissolved in DMSO) containing media were added. After 20 minute incubation (t = 0) TNFα (R&D Systems) was added to the wells to a final concentration of 10 ng/ml. At the indicated times, the wells were washed with PBS and cells were lysed with 350 μl RLT lysis buffer (Qiagen).

### RNA isolation, cDNA synthesis and quantitative real-time PCR

RNA was isolated using RNeasy kit (Qiagen) according to the manufacturer’s instructions. RNA concentrations were measured using NanoDrop ND-1000 instrument (Thermo Fisher Scientific). The cDNA synthesis was performed using 500 ng of total RNA and iScript^™^cDNA Synthesis Kit (Bio-Rad Laboratories, Hercules, USA) in a 20 μl reaction volume. After cDNA synthesis the cDNA was diluted to 1:5. Quantitative real-time PCR was performed from diluted cDNA in iQ^™^ SYBR^®^ Green Supermix (Bio-Rad) using gene specific primers (Table 1) in 20 μl reaction volume. The PCR was performed in iQ5 real-time PCR detection system (Bio-Rad). RPLP0 was used as a housekeeping gene.

**Table 1:** Primers use in quantitative RT-PCR.

Gene	GeneBank Accession	5' Primer	3' Primer	Length
DEC2	NM_030762	TGCTTTACAGAATGGGGAGCGATC	CCCTGGGTGTCCAGCTCTCAAAC	134
IL-1β	NM_000576	TGGCAATGAGGATGACTTGT	GGAAAGAAGGTGCTCAGGTC	237
CCL8	NM_005623.2	TCATGGCAGCCACTTTCAGCC	CCCTGACCCATCTCTCCT	219
CXCL5	NM_002994.4	CCTGCCGCTGCTGTGTTGAG	AGGGAGGCTACCACTTCCACC	137
PER1	NM_002616	CTCCAATCAGGACGCACTTTC	GCTGCCAAAGTATTTGCTTGTG	211
PER3	NM_016831	TGAAGAATCCATCCCATCCTACTG	TATACTGCTGTCGCTGCTTCC	218
DBP	NM_001352	CTTAAGCCCCAGCCAATCATGAAG	CCGCCCGCACCGATATCTG	160
RPLP0	NM_001002	GGCGACCTGGAAGTCCAACT	CCATCAGCACCACAGCCTTC	149

### Plasmids and vectors

DEC2 (NM_030762) cDNA was amplified from human primary fibroblast total cDNA and was inserted into pcDNA3.1 V5 hisA vector (Thermo Fisher Scientific). The following primers were used for DEC2 cDNA amplification: sense 5’-AACGAAGGATCCGCCACCATGGACGAAGGAATTCCTCATTTGCA-3’ and antisense 5’-GGACGCCTCGAGTCAGGGAGCTTCCTTTCCTGGCT-3’.

2 kb part of IL-1β promoter (NG_008851.1) was amplified from Human Genomic DNA (Roche Basel, Switzerland; cat# 11691112001) and inserted into pGL3-Enhancer vector (Promega Corporation, Fitchburg, USA). The following primers were used for amplification: sense 5’-AATTTGGGTACCAATGCTGTCAAATTCCCATTCACCCA-3’ and antisense 5’-TACTTCCTCGAGGGCTGCTTCAGACACTTGAGCA-3’. The constructs were validated by using nucleotide sequencing.

For dual-luciferase assay the control vector was pRL-TK (Promega). Vectors were propagated in competent TOP10 *Escherichia Coli* cells (Thermo Scientific). Ultrapure endotoxin-free plasmid DNA was prepared using NucleoBond^®^ Xtra Midi EF (Macherey-Nagel, Düren, Germany; cat# 740420) according to the manufacturer’s instructions. Plasmid DNA was diluted in a sterile water.

### Transfection

HEK293 cells were seeded on 24-well plates at 4x10^4^ cells per well in 0.5 ml DMEM medium and incubated for 24 h before transfection. For transfection, Fugene HD transfection reagent (Promega, cat# E2311) was used according to manufacturer’s instructions with 500 ng DNA and DNA:Fugene HD ratio of 1:3. All cell manipulations and assays were carried out 48 hours after transfection.

Human primary fibroblasts were transfected using Amaxa Nucleofector II (Lonza) and Amaxa Human Dermal Fibroblast Nucleofector Kit (cat# VPD-1001). Transfection was performed according to manufacturer’s instructions using 4x10^5^ cells, 3 μg DNA and transfection program U-O23. Immediately after transfection cells were seeded on 12-well plates at 1x10^5^ cells per well in 1 ml DMEM medium. All cell manipulations and assays were carried out 24 h after transfection.

### Luciferase assay

Transfection of HEK293 cells was carried out as described using 500 ng of DEC2 expression plasmid or empty control plasmid, 10 ng of reporter plasmid and 1 ng of Renilla luciferase plasmid. Luciferase assay was done using Dual-Luciferase^®^ Reporter Assay System (Promega, cat# E1910) according manufacturer’s instructions 48 h after transfection. Luminescence was measured using Plate CHAMELEON V Multilabel Microplate Reader (Hidex, Turku, Finland).

### siRNA transfection

Human primary fibroblasts were seeded on 24-well plates at 4x10^4^ cells per well in 0.5 ml RPMI-1640 containing antibiotics and 1% FBS. After 12 h, siRNA transfection using RNAiMAX transfection reagent (Thermo Fisher Scientific, cat# 13778) was performed according to manufacturer’s instructions. Briefly, 1.5 μl of Lipofectamine RNAiMAX diluted in 25 μl OPTI-MEM (Thermo Fisher Scientific, cat# 31985) and 15 pmol of ON-Targetplus Human DEC2 (Thermo Fisher Scientific, cat# 79365) siRNA diluted in 25 μl OPTI-MEM were combined and incubated for 5 min at room temperature (RT) after which 50 μl of transfection mix was added per well. After 12 h (t = 0), the media were replaced with RPMI-1640 containing antibiotics, 1% FBS and 10 ng/ml TNFα (R&D Systems) or no added stimulants (negative control). After 10 h, the wells were washed with PBS and lysed with 350 μl RLT lysis buffer (Qiagen).

### Immunofluorescence

Human primary fibroblasts were seeded at 1x10^5^ cells per well on coverslips placed in 12-well plates containing RPMI-1640 supplemented with antibiotics and 1% FBS. Before stimulations the cells were synchronized as described above. For cellular stimulation the media were replaced with RPMI-1640 containing antibiotics and 1% FBS, without or with 10 ng/ml TNFα (R&D Systems). After 24 h cells were washed with PBS and fixed in 4% PFA for 15 min at RT. Fixed cells were permeabilized with 0.1% Triton-X in PBS for 10 min at RT, blocked with 1% BSA-PBS for 1 h at RT, after which slides were incubated with 1 μg/ml rabbit anti-human DEC2 IgG (Santa Cruz Biotechnology, Dallas, USA; cat# sc-32853) or 1 μg/ml non-immune rabbit IgG at 4°C overnight. Next day slides were incubated in 1:100 dilution of Alexa Fluor 568 labeled goat anti-rabbit IgG secondary antibody (Molecular Probes, Leiden, The Netherlands; cat# ab175471) for 1 h at RT, counterstained in 5 μg/ml DAPI and mounted.

### Immunohistochemical staining

Formalin-fixed and paraffin-embedded tissue samples of synovial membranes were cut to 3 μm sections, deparaffinized and rehydrated. Antigens were retrieved in citrate buffer using microwaves (Program AR98C-S30M, MicroMED T/T Mega Histoprocessing Labstation; Milestone Srl, Sorisole, Italy) followed by quenching of endogenous peroxidase in 3% H_2_O_2_ in PBS for 15 min. Sections were incubated in 0.67 mg/ml rabbit anti-human DEC2 IgG (Santa Cruz, cat# sc-32853) at 4°C for overnight. Rabbit IgG at the same concentration was used for negative control staining. Slides were washed with PBS following incubation in biotin-conjugated goat anti-rabbit IgG secondary antibody for 1 h at RT. After washes, slides were incubated for 1h at RT in freshly prepared avidin–biotin–peroxidase complexes (Vector Laboratories, Burlingame, USA; Vectastain Elite ABC kit). Color was developed using H_2_O_2_ and DAB. Between each step slides were washed at least three times with PBS. Finally, slides were dehydrated, counterstained in haematoxylin and coverslips were mounted using Mountex (Histolab, Västra Frölunda, Sweden).

### Statistical analysis

The data of IL-1β or DEC2 expression after TNFα stimulation was analyzed with repeated measures ANOVA. Significance was tested using Bonferroni. Reported p-value is difference of TNFα stimulation and mock group. The means of the experiments with two independent samples were tested using student’s t-test. Tests were performed with SPSS 15.0 for Windows (SPSS Inc. Chicago, IL). All results are expressed as mean ± SEM unless otherwise stated in the figure legend.

## Results

### TNFα stimulates the expression of DEC2 but not DEC1

**Fig 1:**
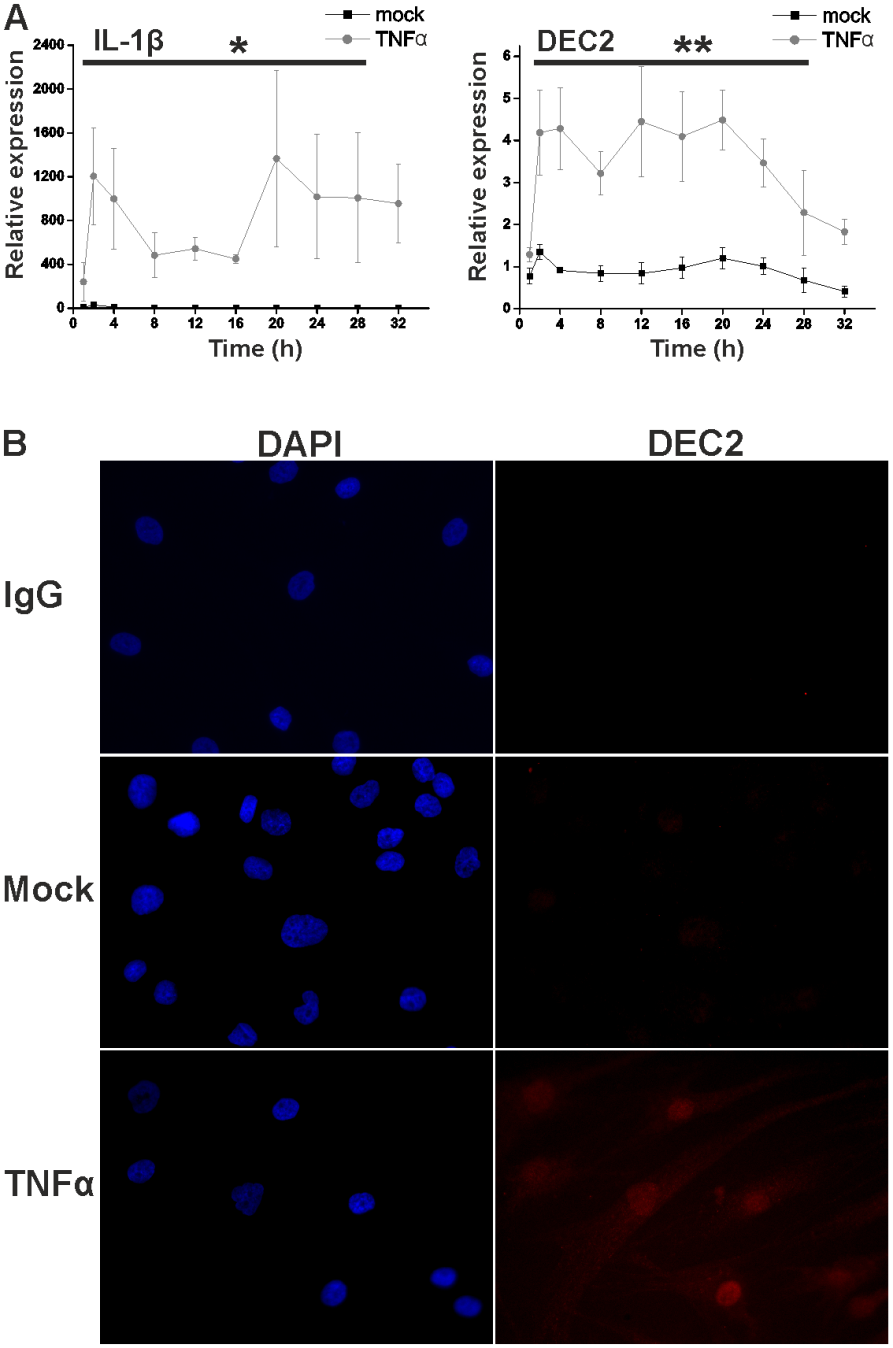
The response of human fibroblasts to TNFα stimulation. After 24h incubation in medium containing 1% FBS, the cells were incubated in fresh medium containing TNFα (10ng/ml) or PBS. **A**, mRNA expression of IL-1β and DEC2 after the stimulus. Samples were collected at indicated time points. Values represent means ± SEM of four different experiments performed in duplicate. * p < 0.05, ** < 0.001, repeated measures ANOVA. **B**, After the stimulation fixation and blocking, the cells were incubated overnight with DEC2 antibody or rabbit IgG at 4°C followed by secondary fluorescent antibodies (red) and nuclear counterstain with DAPI (blue). Induction and nuclear localization of DEC2 protein is evident after TNFα stimulation.

To study the eventual TNFα effects on DEC1 and DEC2, synovial fibroblasts were synchronized by serum starvation after which they were stimulated without or with 10 ng/ml TNFα. TNFα upregulates IL-1β, which was therefore used as a positive control in TNFα stimulation experiments. TNFα-mediated increase of IL-1β (p < 0.05, F 9.6, df between groups 1,6) confirmed that the stimulation was successful (Fig 1A). Samples collected at 1, 2 and 4 hours and then every 4 hours up to 32 hours were analyzed for DEC1 (which was not changed, data not shown) and DEC2 mRNA (Fig 1A). TNFα increased DEC2 expression 4-fold (p < 0.001, F 50.6, df between groups 1,6) already at 2 hours and this effect was maintained until the 32 hour time point. The effect of TNFα on DEC2 was also shown by immunofluorescence staining of TNFα stimulated synovial fibroblasts (Fig 1B). DEC2 was increased also at the protein level and mainly localized in nuclei of TNFα stimulated cells.

**Fig 2:**
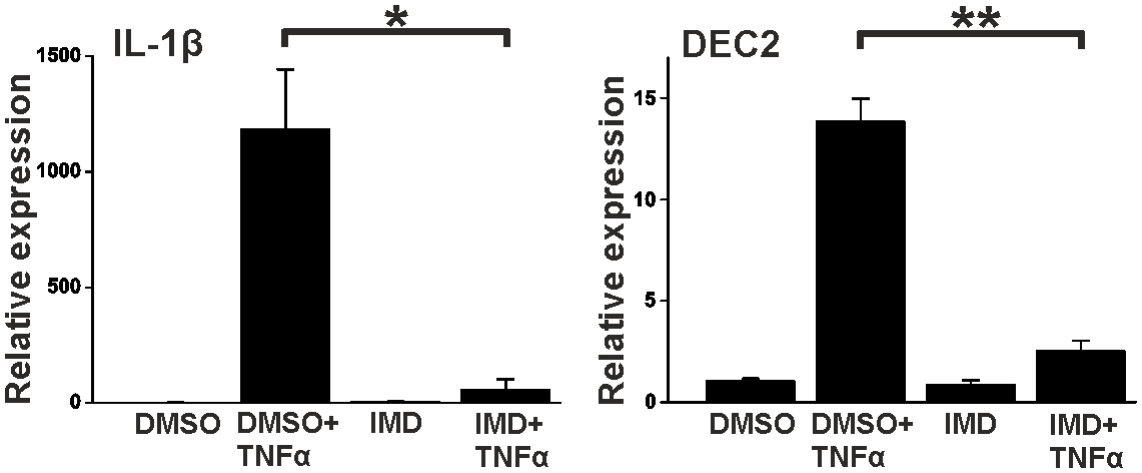
NF-κB pathway regulates the expression of DEC2. After 24h incubation in medium containing 1% FBS, human fibroblasts were incubated in fresh medium containing 1 μM IMD-0354 (abbreviated IMD) diluted in DMSO. After 20 min incubation cells were stimulated with TNFα (10ng/ml) or PBS for 16h. IL-1β and DEC2 were measured using real time PCR. Values represent means ± SEM of three different experiments performed in duplicate. * p < 0.05, ** p < 0.001, t-test.

To test if TNFα effect on DEC2 expression is mediated by NF-κB pathway, synovial fibroblasts were stimulated as above but first after 20 min pretreatment with 1 μM IKK-2 inhibitor IMD-0354. Successful inhibition was confirmed by studying the expression of IL-1β (p < 0.05, t-value 4.1, df 4) (Fig 2). Samples collected at 16 hours of stimulation (the highest peak of DEC2 expression) were analyzed for DEC2 mRNA. IMD-0354 significantly (p < 0.001, t-value 9.0, df 4) inhibited the TNFα-induced DEC2 expression. The 15-fold expression was reduced to only 2-fold when NF-κB pathway was inhibited (Fig 2).

### DEC2 overexpression stimulates IL-1β expression in HEK293 cells and in human fibroblasts

**Fig 3:**
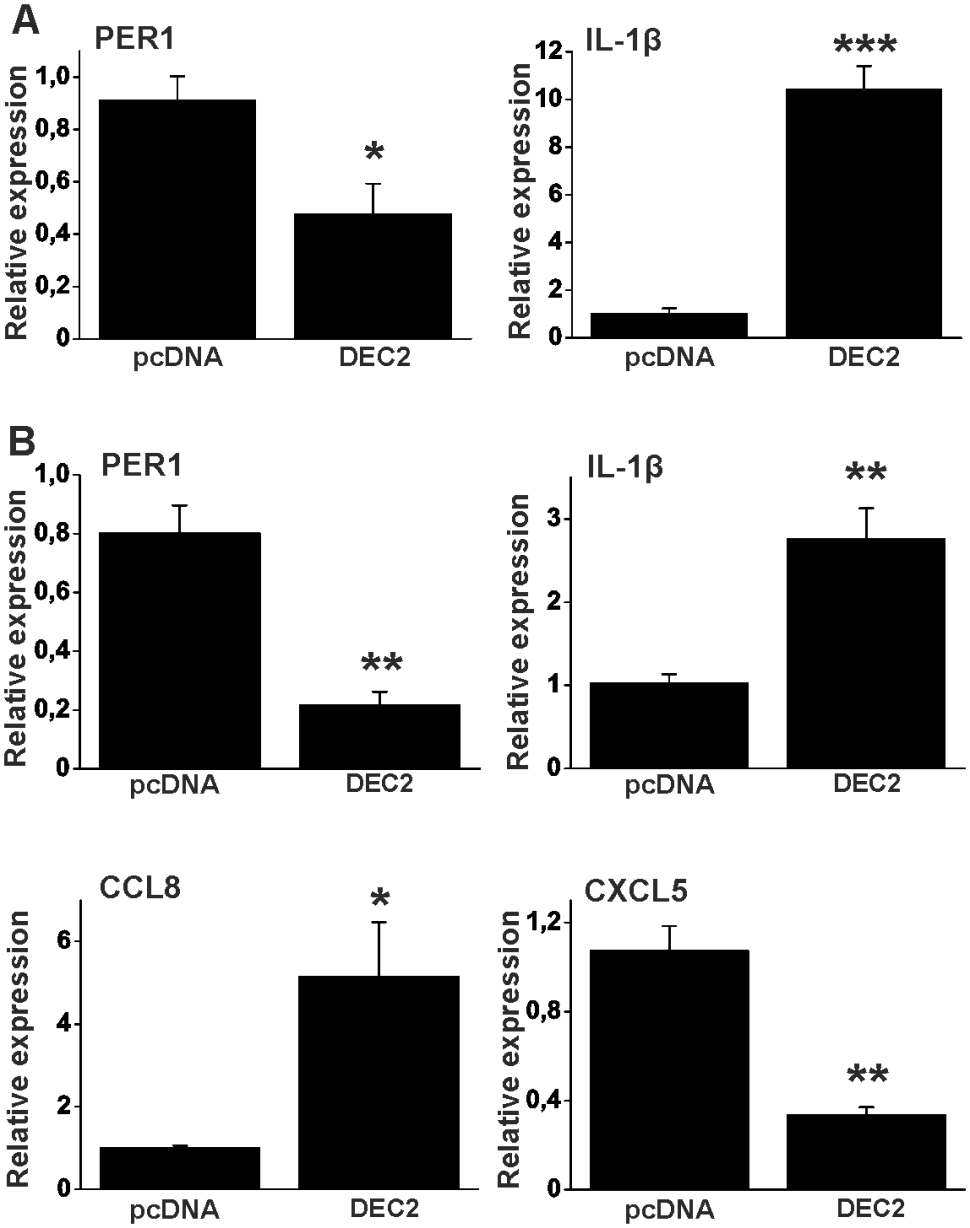
DEC2 regulates PER1 and inflammatory factors. **A**, HEK293 cells were transfected with empty vector (pcDNA) or DEC2 containing vector. After 48h samples were analyzed using RT-qPCR. Values represent means ± SEM of four different experiments performed in duplicate. * p < 0.05, *** p < 0.001, t-test. **B**, Human fibroblasts were transfected with empty vector (pcDNA) or DEC2 containing vector. After 24h samples were analyzed using RT-qPCR. Values represent means ± SEM of three different experiments performed in triplicate. * p < 0.05, ** p < 0.01, t-test.

Because TNFα increases the expression of DEC2 and IL-1β, it was hypothesized that DEC2 itself might contribute to the upregulation of IL-1β. To test this hypothesis, DEC2 gene was cloned and overexpressed in HEK293 cells. DEC2 downregulates Per1 [10], which was therefore used as a positive control of DEC2 function in HEK293 cells (Fig 3A) and in synovial fibroblasts (Fig 3B). Both experiments demonstrated that DEC2 significantly reduced the expression of PER1 (p < 0.05, t-value 4.2, df 4 in HEKs and p < 0.01, t-value 5.6, df 4 in fibroblasts). In addition to this, DEC2 inhibited the expression of DBP and PER3 (not shown) confirming that its overexpression may contribute to the reduction of clock output genes after TNFα stimulation. DEC2 overexpression increased the expression of IL-1β mRNA 8-fold (p < 0.001, t-value 9.6, df 4) in HEK293 cells (Fig 3A) and 3-fold (p < 0.01, t-value 4.6, df 4) in human synovial fibroblasts (Fig 3B) compared to empty vector controls. Because CCL8 and CXCL5 are regulated by components of the circadian clock [19, 20], we investigated their regulation by DEC2 in human cells. Indeed they were significantly (p < 0.05, t-value 3.5, df 4 for CCL8 and p < 0.01, t-value 6.4, df 4 for CXCL5) regulated by DEC2 in human synovial fibroblasts (Fig 3B).

### DEC2 overexpression further increases TNFα responses

**Fig 4:**
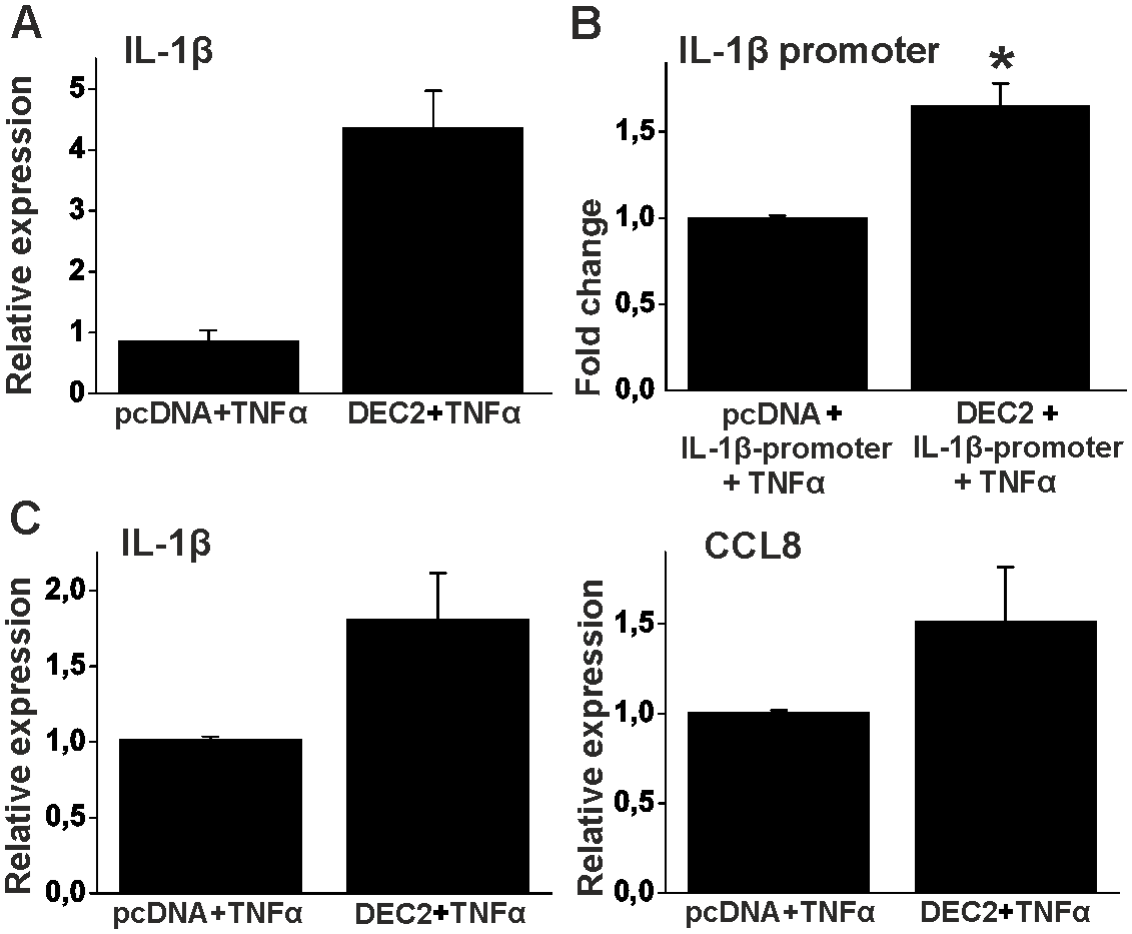
DEC2 potentiates the effect of TNFα. HEK293 cells were transfected with empty vector (pcDNA) or DEC2 containing vector and stimulated with TNFα (10ng/ml) for 24h (A) or 8h (B). **A**, IL-1β mRNA levels analyzed using RT-PCR. **B**, IL-1β promotor activity was analyzed using luciferase assay. Expression levels and promoter activities are compared to TNFα stimulated samples transfected only with empty pcDNA vector. Values represent means ± SD in panel A and ± SEM in panel B. Panel A represents a single experiment performed in triplicate and panel B three different experiments performed in duplicate. * p < 0.05, t-test. **C**, Human fibroblasts were transfected with empty vector (pcDNA) or DEC2 containing vector. Cells were stimulated with TNFα (10ng/ml) for 24h. Samples were analyzed by using RT-qPCR. Values represent means ± SD of single experiments performed in duplicate.

TNFα induces the expression of IL-1β both in human fibroblasts (Fig 1A) and in HEK293 cells (hundred fold; data not shown). It may well be that DEC2 only induces IL-1β in unstimulated cells. Thus, we wanted to test the effect of DEC2 during TNFα stimulus. Overexpression of DEC2 in HEK293 cells increased IL-1β mRNA levels in response to TNFα 4-fold (Fig 4A). Accordingly, DEC2 also increased TNFα mediated IL-1β promoter activity (p < 0.05, t-value 4.3 df 4) suggesting that this increase results in part from increased transcription (Fig 4B). This effect was also true in human fibroblasts. Overexpression of DEC2 also in these cells increased IL-1β and CCL8 mRNA levels in response to TNFα (Fig 4C).

### DEC2 silencing decreases TNFα responses

**Fig 5:**
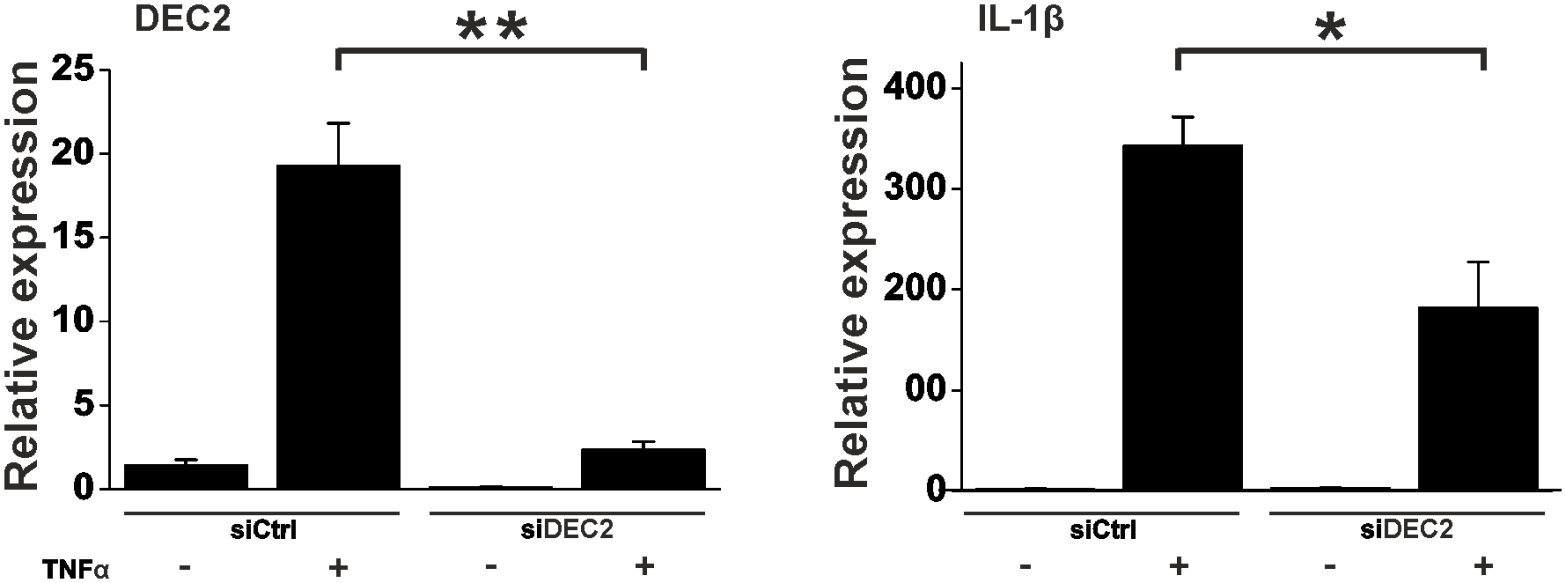
Silencing of DEC2 in human fibroblasts reduces IL-1β expression after TNFα stimulation. After 12h incubation in medium containing 1% FBS, DEC2 was silenced using Thermo Scientific On-Targetplus SMART pool DEC2 siRNA. 12h after siRNA transfection, medium was replaced with fresh medium containing TNFα (10ng/ml) or PBS. Samples were collected 10h after stimulation. Values represent means ± SEM of three different experiments performed in duplicate. * p < 0.05, ** p < 0.005, t-test.

If the results from overexpression experiments were true, silencing of DEC2 should lead to decrease of IL-1β expression. To verify the results, silencing of DEC2 using siRNA was performed. Indeed silencing of DEC2 (p < 0.005, t-value 6.5, df 4) declined the IL-1β increase (p < 0.05, t-value 3.0, df 4) in response to TNFα in human fibroblasts (Fig 5).

### DEC2 protein is abundant in the synovial membrane in RA

**Fig 6:**
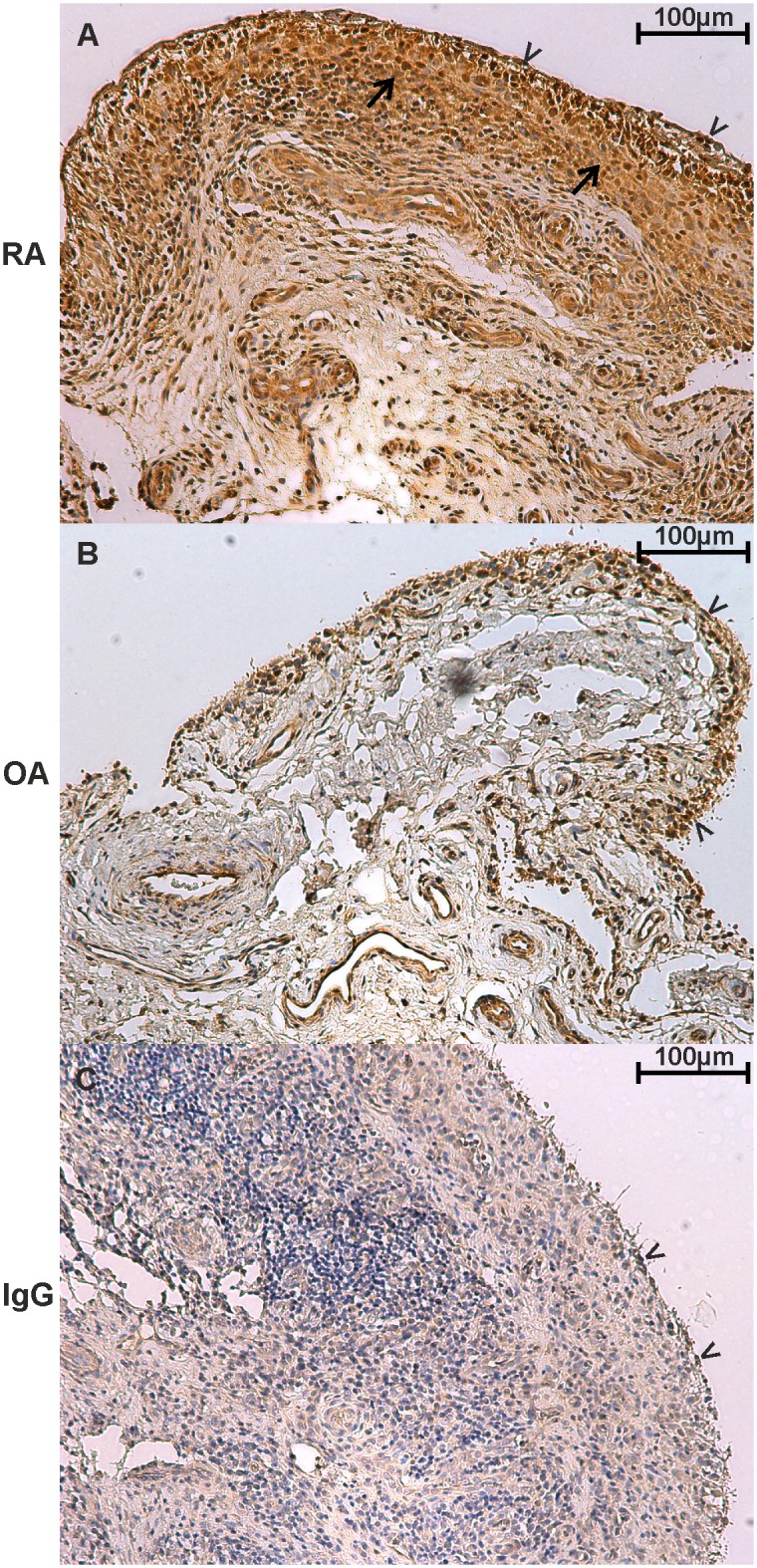
Abundant DEC2 expression in synovial membrane samples from RA patients. Tissue sections were incubated with DEC2-speficic antibody (0.67 μg/ml) at 4°C overnight. Negative staining control of a RA sample was stained using normal rabbit IgG at the same concentration. After ABC staining, the slides were counterstained with hematoxylin. Arrowheads point to the lining cells which exhibit a strong staining reaction. DEC2 does not only localize into the nucleus but it is also abundant in the cytoplasm (arrows).

Due to the above described *in vitro* effects of TNFα on upregulation of DEC2, RA and OA synovial tissues were immunostained for the presence of DEC2. DEC2 staining was much more intense and extensive in RA synovitis tissue (Fig 6A) than in more mildly inflamed OA synovial tissue samples (Fig 6B). Negative staining controls confirmed the specificity of the staining (Fig 6C).

## Discussion

BMAL1/CLOCK heterodimer is the major component of the molecular pacemaker responsible for the normal homeostatic circadian rhythm. Its major counter-regulators are PER1-3 and CRY1-2, which in various complexes cyclically oscillate in a fashion reciprocal to that of the BMAL1/CLOCK, regulating the length of the circadian cycle. However, yet another regulatory paralogue pair exists in the negative feedback loop controlling unconstrained and continued effect of the BMAL1/CLOCK [21]. Due to the apparently disturbed circadian rhythm in RA and the upregulated ARNTL2, NPAS2 but paradoxically downregulated DBP and PER3 mRNA expression after TNFα stimulations, the clock counter-regulators DEC1 and DEC2 were analyzed in resting and TNFα stimulated human synovial fibroblasts. Fibroblast was selected as the major target cell because it is an important cellular component of synovial stromal connective tissue, erosive pannus and synovial lining, in which fibroblast-like type B lining cells together with macrophage-like type A lining cells form its two cellular components [22]. Because the central circadian pacemaker at SCN regulates the peripheral clocks in all peripheral cells, fibroblast should in principle be as good indicator of the regulation of the clock components as any other cell type. It was found that the pro-inflammatory cytokine TNFα stimulates DEC2 at both mRNA and protein level in a NF-κB-dependent manner in cultured human synovial fibroblasts. Further studies focused on DEC2 because its paralogue DEC1 was not affected by TNFα.

IL-1β displays circadian rhythm in circulation and its expression is rhythmic in fibroblasts [23, 24]. Thus, we wanted to test if DEC2 by itself without upstream TNFα has some independent effects on IL-1β. DEC2 was cloned and first transfected to HEK293 cells. It was shown that IL-1β is increased in both DEC2- and TNFα-dependent manner in HEK293 cell. This was then confirmed for IL-1β via transcription and promoter activation and also for some other pro-inflammatory cytokines by overexpression of DEC2 in human synovial fibroblasts. Thus, TNFα exerts its inflammatory effects in part through DEC2 suggesting that this component of the molecular clock participates in the regulation of inflammatory responses also in human cells. This conclusion was further confirmed by silencing DEC2 with siRNA that significantly decreased TNFα-induced IL-1β expression. Although silencing of DEC2 was quite effective, its effect on TNFα-induced IL-1β was only partial. This suggests that the upregulation of IL-1β by TNFα is only partially DEC2-dependent. There are several signaling pathways and transcription factors that are known to be activated after TNFα stimulus [25]. Thus, it is not surprise that DEC2 is not completely responsible for the regulation of IL-1β.

NF-κB pathway is involved in the transcriptional activation of a vast number of inflammatory and apoptotic machinery genes in response to TNFα [25]. DEC2 is involved in the control of apoptosis in cancer cells [26]. Thus, the hypothesis was that TNFα induced DEC2 expression is mediated via the NF-κB pathway. Indeed, the induction of DEC2 was almost completely suppressed by the inhibition of IKK-2.

DEC2 protein levels were much higher in RA synovial membrane than in OA synovial membrane. This is in accordance with the higher degree of inflammation and TNFα production in RA compared to that of OA [27]. The high impact of TNFα on the pathomechanisms of RA is supported by the overall effectiveness of anti-TNF drugs in the clinical setting [28]. Many different mechanisms of action of anti-TNF drugs have been suggested, such as diminished expression of vascular endothelial adhesion molecules and therefore diminished recruitment of inflammatory leukocytes to synovitis tissue [29]. The present findings suggest that TNFα and anti-TNF drugs may also affect disease activity and progress via regulation of the circadian clock, which further participates in the regulation of immune responses and fatigue [30].

It can be concluded that DEC2 is aberrantly expressed in RA tissue, it is induced by TNFα and not only affects the expression of genes belonging to molecular clock but also significantly impacts on the expression of IL-1β as well as other inflammatory genes.
